# Supplementary material for: Li-ion storage properties of two-dimensional titanium-carbide synthesized via fast one-pot method in air atmosphere
Source: Nat Commun. 2021 Aug 24;12:5085. doi: 10.1038/s41467-021-25306-y (PMC8385058; doi:10.1038/s41467-021-25306-y)
Supplement: Supplementary file 1 — Supplementary Information [file 41467_2021_25306_MOESM1_ESM.pdf]

Supplementary Materials for

Li-ion storage properties of two-dimensional titanium-carbide synthesized via fast one-pot method in air atmosphere

Guoliang Ma[1†], Hui Shao[2,3†], Jin Xu[4], Ying Liu[1*], Qing Huang[5,6], Pierre-Louis Taberna[2,3], Patrice Simon[1,2,3*], Zifeng Lin[1*]

[1] College of Materials Science and Engineering, Sichuan University, Chengdu, 610065, China

[2] CIRIMAT, Université de Toulouse, CNRS, France

[3] Réseau sur le Stockage Electrochimique de l'Energie (RS2E), FR CNRS n°3459

[4] School of Machine Engineering, Dongguan University of Technology, Dongguan, 523808, China

[5] Engineering Laboratory of Advanced Energy Materials, Ningbo Institute of Materials Technology and Engineering, Chinese Academy of Sciences, Ningbo, Zhejiang 315201, China

[6] Qianwan Institute of CNiTECH, Zhongchuangyi Road, Hangzhou Bay District, Ningbo, Zhejiang, 315336, China

*Correspondence to:

Prof. Patrice Simon, E-mail: simon@chimie.ups-tlse.fr

Prof. Zifeng Lin, E-mail: linzifeng@scu.edu.cn

Prof. Ying Liu, E-mail: liuying5536@scu.edu.cn

[†] These authors contributed equally to this work.

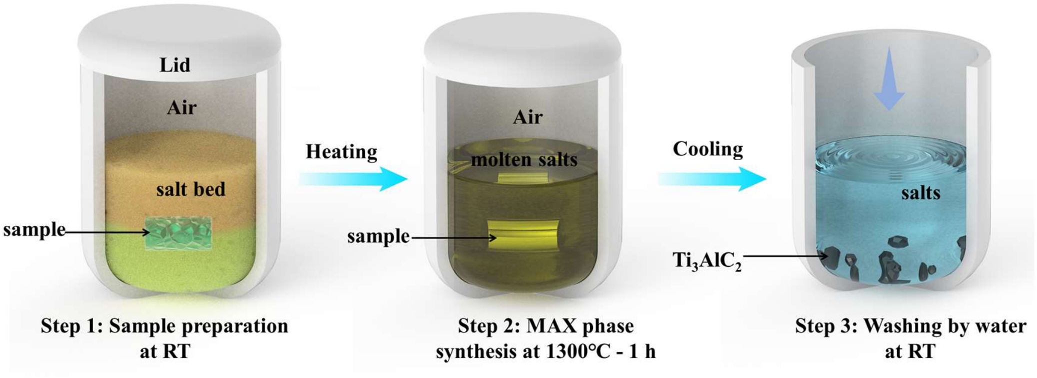

Supplementary Fig. 1. Molten salt shielded synthesis (MS^3) of MAX phase. Schematic diagram of the molten salt shielded synthesis (MS^3) of Ti_3AlC_2-MAX in an air atmosphere. Sample pellet is made up of 1.45 g Ti, 0.33 g Al, 0.23 g C (3:1.2:1.9 by molar ratio) powders mixed with 1.77 g NaCl and 2.25 g KCl salts.

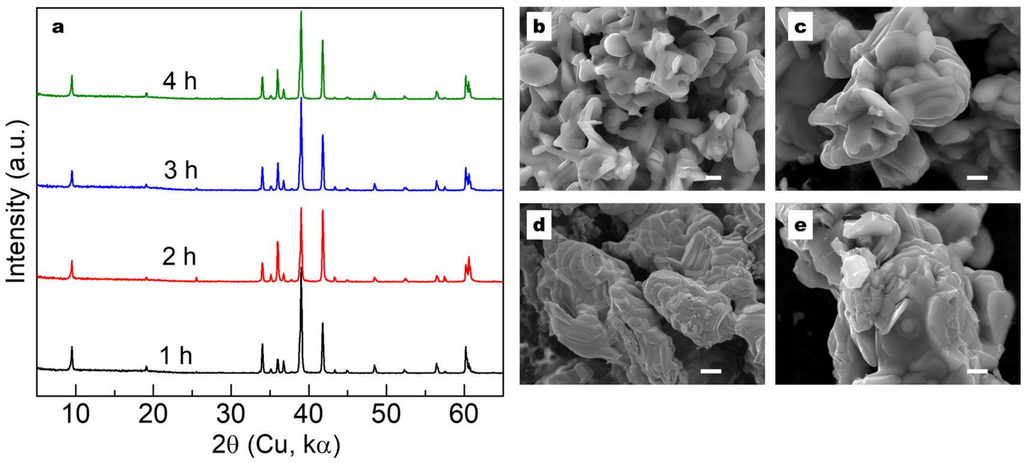

Supplementary Fig. 2. Characterizations of MS^3-Ti_3AlC_2 MAX phase. (a) XRD patterns and **(b-e)** SEM images of MS^3-Ti_3AlC_2 at 1300 ºC with reaction time of **(b)** 1 h; **(c)** 2 h; **(d)** 3 h; **(e)** 4 h, scale bars are 1μm.

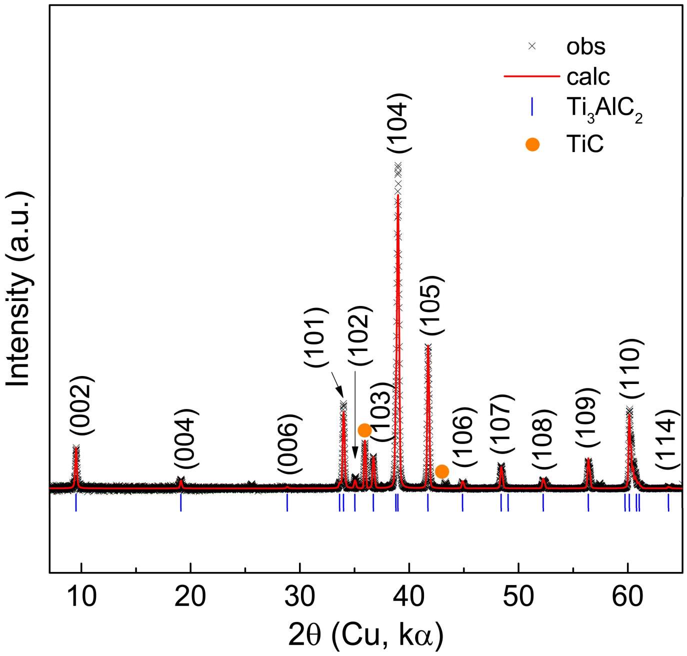

Supplementary Fig. 3. XRD pattern and Rietveld refinement of MS^3-Ti_3AlC_2 sample.

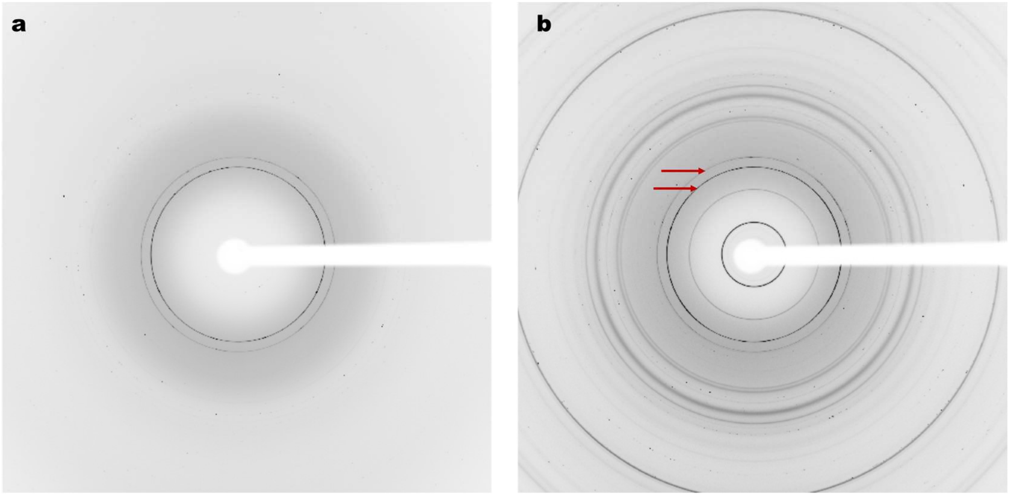

Supplementary Fig. 4. Synchrotron X-ray diffraction analysis of one-pot synthesized $Ti_3C_2T_x$ MXene. 2D synchrotron XRD patterns of **(a)** sample holder (blank test), and **(b)** $Ti_3C_2T_x$ prepared by 10 minutes etching at 700°C. The red arrows point to the diffraction rings of the sample holder.

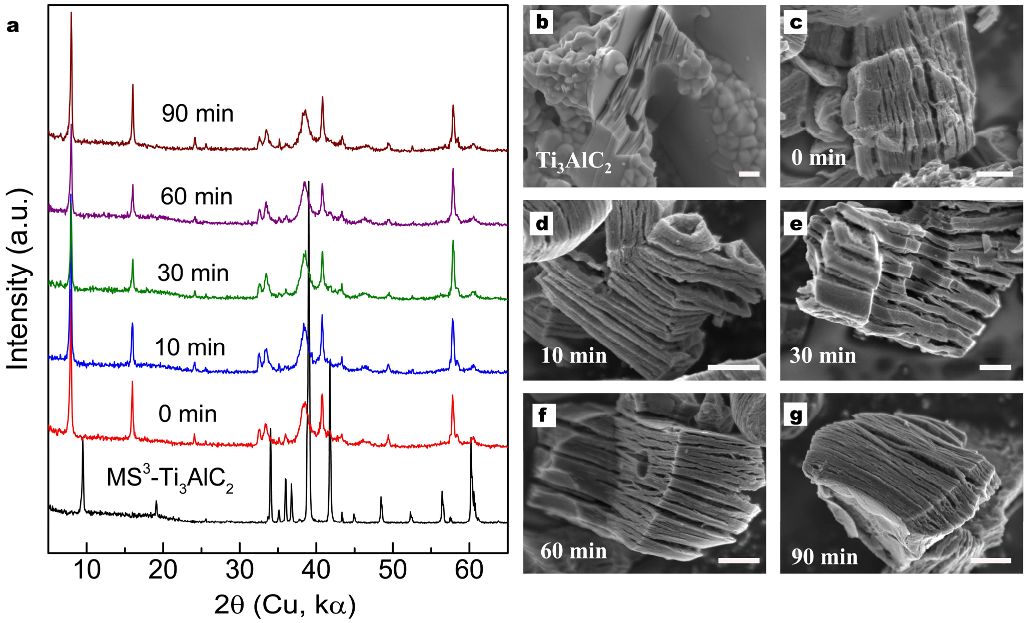

Supplementary Fig. 5. Characterizations of Ti_3AlC_2 MAX phase and one-pot synthesized $Ti_3C_2T_x$ MXene. (a) XRD patterns and **(b-g)** SEM images of molten salt prepared Ti_3AlC_2 and one-pot synthesized $Ti_3C_2T_x$ MXene with 0, 10, 30, 60, 90 minutes etching reaction at 700 ^{o}C respectively, scale bars are 0.5 µm.

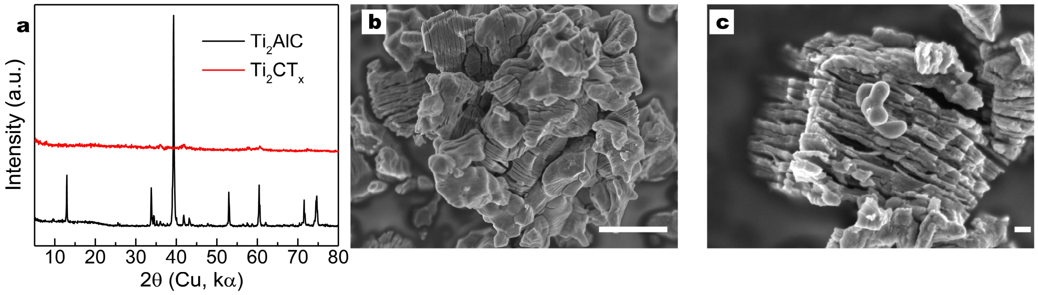

Supplementary Fig. 6. Characterizations of one-pot synthesized Ti_2CT_x MXene. **(a)** XRD patterns of MS^3-Ti_2AlC and one-post synthesized Ti_2CT_x; **(b)** SEM images of Ti_2CT_x MXene at scale bars of **(b)** 1 μm, **(c)** 100 nm.

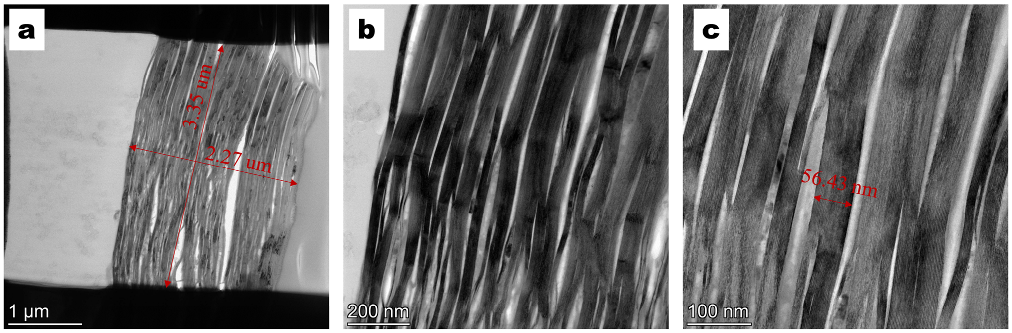

Supplementary Fig. 7. High-resolution TEM images of cross-sectional view of a $Ti_3C_2T_x$ MXene particle cut by FIB. Scale bars are **(a)** 1 μm, **(b)** 200 nm, **(c)** 100 nm, respectively. The particle size is about 2.27 μm in width and 3.35 μm in length. The $Ti_3C_2T_x$ MXene ribbon is measured with 56.43 nm in stacking thickness.

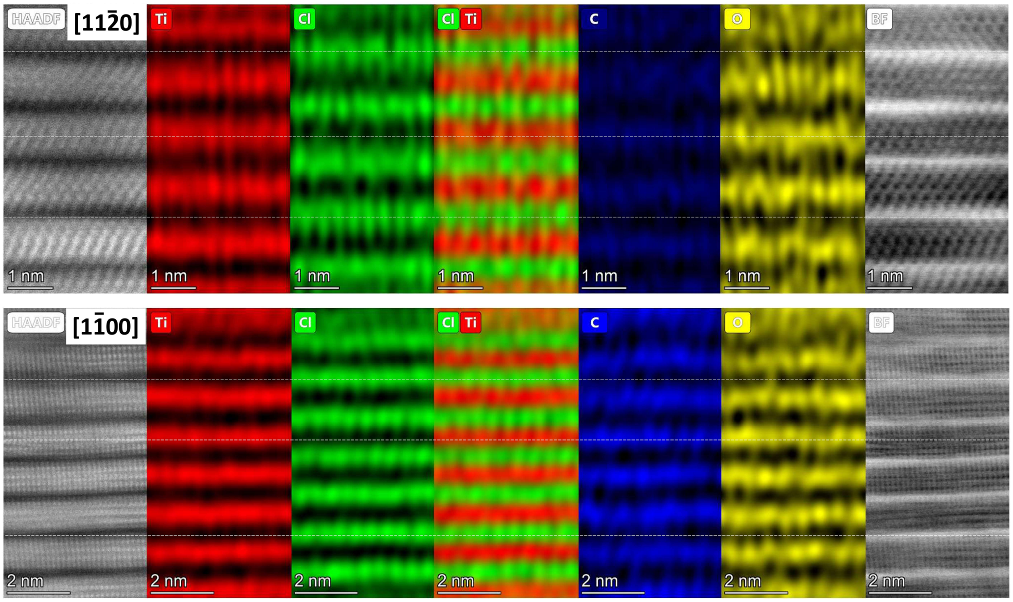

Supplementary Fig. 8. Atomic structural analysis of $Ti_3C_2T_x$. Atomic scale EDS mapping of $Ti_3C_2T_x$ MXene along [11$\bar{2}$0] and [1$\bar{1}$00] projections.

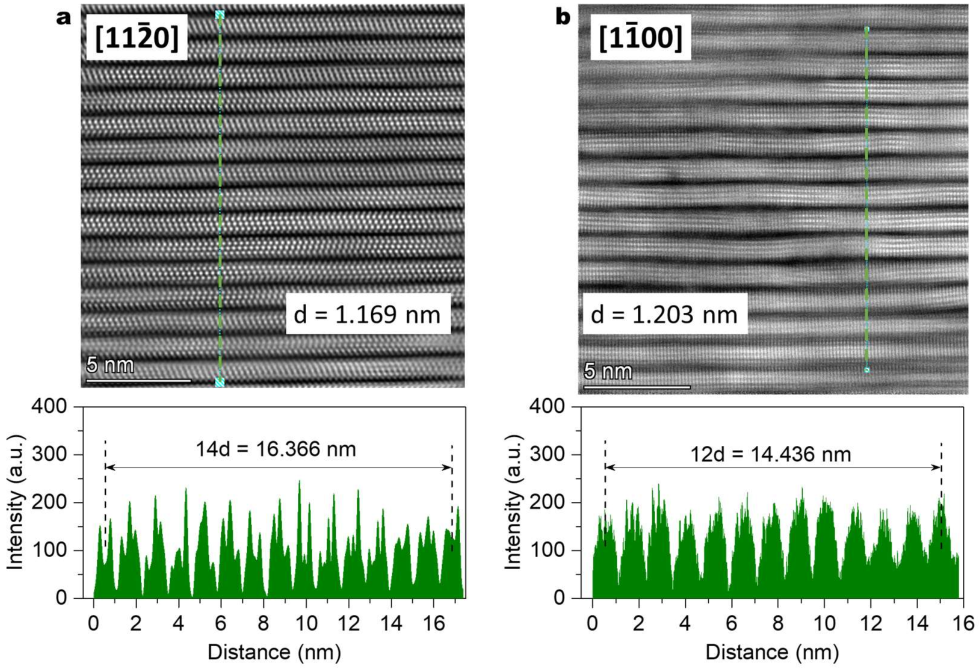

Supplementary Fig. 9. **Atomic structural analysis of interlayer spacing of $Ti_3C_2T_x$**. HAADF–STEM images and corresponding HAADF intensity profiles across the $Ti_3C_2T_x$ MXene layers along the dashed line along **(a)** [11$\bar{2}$0] and **(b)** [1$\bar{1}$00] projections.

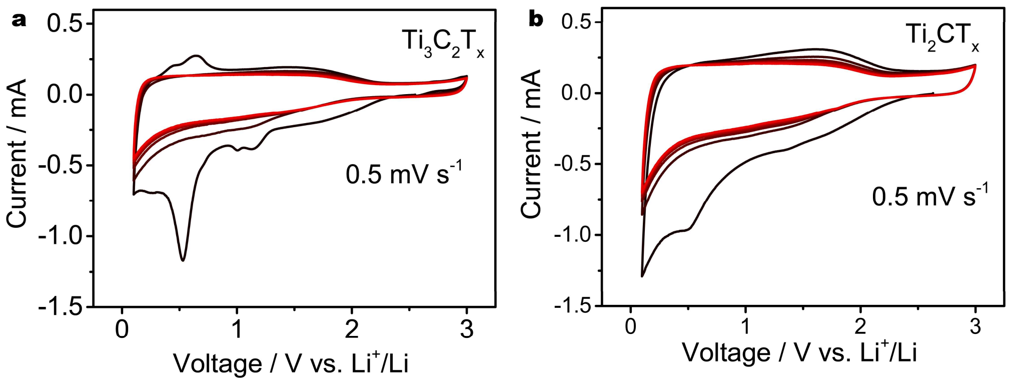

Supplementary Fig. 10. **Electrochemical characterizations of one-pot synthesized MXenes.** Initial six cyclic voltammetry cycles of **(a)** $Ti_3C_2T_x$ and **(b)** Ti_2CT_x electrode at a scan rate of 0.5 mV s^{-1}. The reduction peaks observed at about 0.6 V vs Li^+/Li of both electrodes correspond to the formation of SEI layer[1], and the reduction peak at 1.2V of $Ti_3C_2T_x$ electrode is assumed to be linked with irreversible reaction on defective Ti site or under-coordinated Ti. The oxidation peak observed at the first cycle at 0.5 V of $Ti_3C_2T_x$ electrode is not clear and under investigation currently.

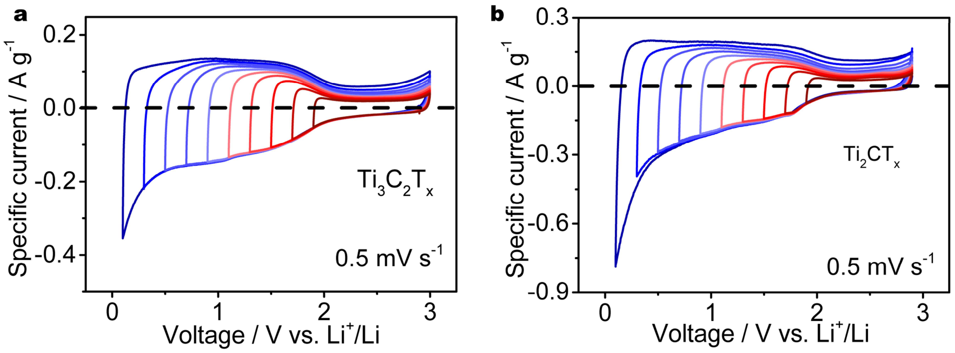

Supplementary Fig. 11. **Electrochemical characterizations of one-pot synthesized MXenes.** Cyclic voltammetry curves with various negative cut-off potentials of **(a)** $Ti_3C_2T_x$ and **(b)** Ti_2CT_x electrode at a scan rate of 0.5 mV s^{-1}.

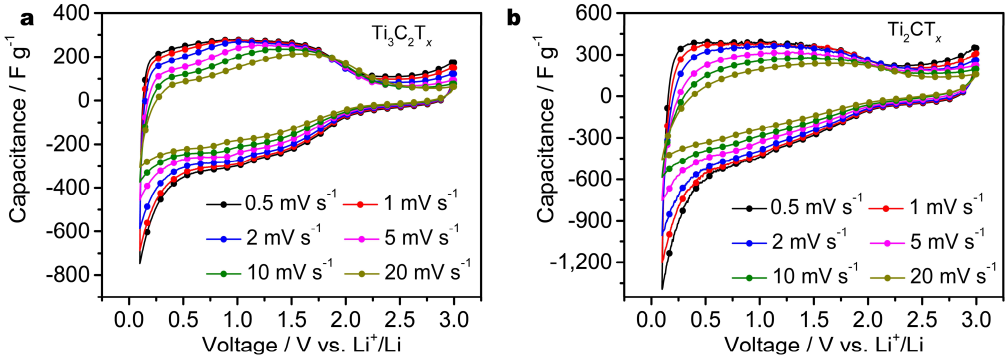

Supplementary Fig. 12. **Electrochemical characterizations of one-pot synthesized MXenes.** Cyclic voltammetry profiles of **(a)** $Ti_3C_2T_x$ and **(b)** Ti_2CT_x electrodes at scan rates from 0.5 to 20 mV s^{-1}.

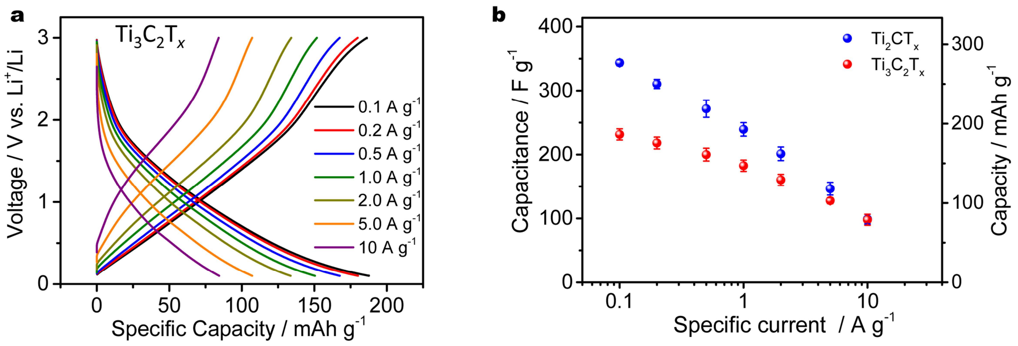

Supplementary Fig. 13. **Electrochemical characterizations of one-pot synthesized MXenes. (a)** Galvanostatic voltage profiles of $Ti_3C_2T_x$ at specific currents range from 0.1 to 10 A g^{-1}; **(b)** Comparison of specific capacity vs. specific currents, the data in present figure are mean value ± standard deviation of three electrochemical cells.

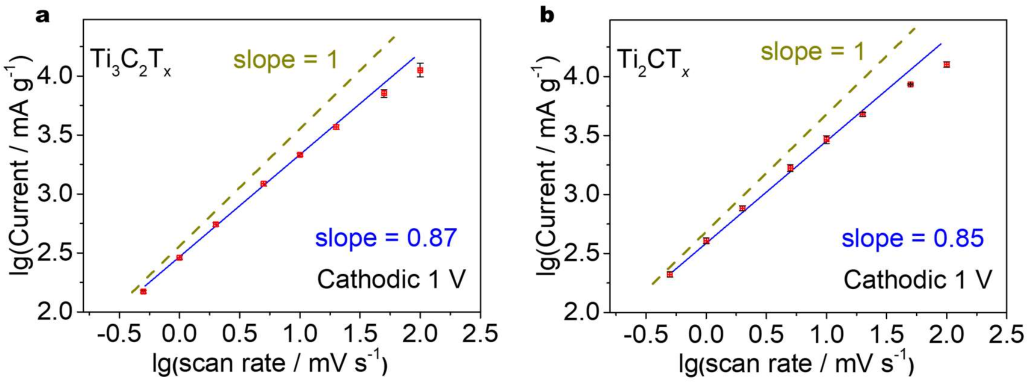

Supplementary Fig. 14. **Kinetic analysis of MXene electrodes.** *b*-value determination by the plot of log(scan rate) vs log(current). The data in present figures are mean value ± standard deviation of three electrochemical cells.

Supplementary Table 1 Time consumption of each step in MXene synthesis process, **RT**: room temperature; **MT**: maximum temperature, 1300 ºC for $Ti_3C_2T_x$, 1000 ºC for Ti_2CT_x.

MXenes preparation	From RT to MT (minutes)	Holding at MT (minutes)	From MT to 700 ºC (minutes)	Holding at 700 ºC (minutes)	From 700 ºC to RT (minutes)	Total (minutes)
$Ti_3C_2T_x$	150	60	120	0-90	120	450-540
Ti_2CT_x	100	60	60	10	120	350

Supplementary Table 2 Li^+ storage capacities of $Ti_3C_2T_x$ electrode at different scan rates.

Scan rate/ mV s^{-1}	Capacitance/ F g^{-1}	Capacity / mAh g^{-1}	Time / min (C-rate)	Coulombic efficiency / %
0.5	204	164	96.7 (0.6)	98.5
1	192	155	48.3 (1.2)	98.9
2	177	142	24.2 (2.5)	99.1
5	157	126	9.7 (6.2)	99.3
10	141	113	4.8 (12.4)	99.5
20	123	99	2.4 (24.8)	99.7
50	99	80	0.97 (62.1)	99.9
100	79	64	0.48 (124.1)	100.1

Supplementary Table 3 Li^+ storage capacities of Ti_2CT_x electrode at different scan rates.

Scan rate/ mV s^{-1}	Capacitance/ F g^{-1}	Capacity / mAh g^{-1}	Time / min (C-rate)	Coulombic efficiency / %
0.5	318	256	96.7 (0.6)	97.6
1	299	241	48.3 (1.2)	97.8
2	276	223	24.2 (2.5)	98.3
5	236	190	9.7 (6.2)	98.6
10	204	164	4.8 (12.4)	99.3
20	169	136	2.4 (24.8)	99.8
50	126	102	0.97 (62.1)	99.4
100	95	76	0.48 (124.1)	98

Supplementary Reference

1 Li, Y. *et al.* A general Lewis acidic etching route for preparing MXenes with enhanced electrochemical performance in non-aqueous electrolyte. *Nature Materials* **19**, 894-899 (2020).
